# The mechanical behavior of as received and retrieved nickel titanium orthodontic archwires

**DOI:** 10.1186/s40510-018-0251-z

**Published:** 2019-01-07

**Authors:** Luca Lombardo, Giorgia Toni, Valentina Mazzanti, Francesco Mollica, Giorgio Alfredo Spedicato, Giuseppe Siciliani

**Affiliations:** 1 Postgraduate School of Orthodontics, University of Ferrara, Via Borsari, 46, 44121 Ferrara, Italy; 2 Department of Engineering, University of Ferrara, Ferrara, Italy

**Keywords:** Retrieved NiTi archwire, Fatigue, Mechanical behavior

## Abstract

**Objectives:**

The aim of this study is to investigate and compare the characteristics of as received and retrieved NiTi archwires at a constant temperature by plotting their load/deflection graphs and quantifying three parameters describing the discharge plateau phase.

**Materials and methods:**

Two hundred four NiTi archwires, traditional and heat-activated, of various cross sections, were obtained from 5 different manufacturers. Specimens prepared from the selected wires were subjected to a three-point bending test where 92 were retrieved through an in vivo retrieval protocol (crowding group C1 and group C2), 56 went through an in vitro retrieval protocol, and 56 were as received. The in vitro retrieval protocol was performed by a gear motor connected to a stainless steel support that performed fatigue cycles to the bent wires in artificial saliva. The load/deflection graphs of as received and retrieved wires were described through three parameters and the results were analyzed with classification and regression trees (CART) and analysis of variance (ANOVA).

**Results:**

Statistically significant differences between as received and retrieved wires were found only for the parameter plateau slope which represents the constancy of force expressed by the wire.

**Conclusions:**

The aging of NiTi archwires influences the force constancy expressed. The behavior of the wires changes depending on the size, brand, and type of retrieval protocol. In terms of performance, the poorest is represented by all wires retrieved in vitro and in vivo group C2 (moderate to severe crowding).

## Introduction

The use of nickel titanium archwires as an initial wire in the leveling and alignment stages of treatment has increased significantly since their introduction in the 1970s [1]. Their use is due to their properties of shape memory and superelasticity [2]. Both these effects are related to the ability of NiTi archwire to easily transform to and from a martensitic phase. Transformation can occur by means of stress or temperature changes [3]. The NiTi mechanical properties have facilitated their clinical use and the low load deflection ratio for this alloy, over a wide range of deformation, has contributed to the establishment of long intervals between appointments and the reduction of the required screening visits. It has been recently demonstrated, though, that temperature does affect permanently the NiTi mechanical behavior [4]. Although much research has focused on the study of the mechanical properties, there is a scarcity of information on in vivo aged orthodontic wires mechanical properties [5, 6]. The majority of the few published reports analyzing in vivo aged wires has focused on the study of corrosion resistance and surface morphology [5, 7].

## Materials and methods

Two hundred four types of NiTi archwires were tested, of which 94 were classed as traditional NiTi and 110 as heat-activated wires. The wires were circular and rectangular in cross section and had a diameter of 0.014, 0.016, and 0.019X0.025 in. The archwires were provided by Dentaurum (Ispringen, Germany), Forestadent (St Louis, MO), Ormco (Orange, Calif), G&H (Franklin, Ind), and American Orthodontics (Sheboygan, Wis) as summarized in Table 1. The wires were divided in three groups: the IN VIVO group, wires retrieved from patients treated at the Orthodontic Clinic, Orthodontic School of Ferrara, University of Ferrara, Italy and collected during regular recall visits; the IN VITRO group, wires retrieved in the laboratory of the engineer department at the University of Ferrara; and the AS RECEIVED group, wires as sent by the companies.

**Table 1:** Orthodontic wires tested

Manufacturer	Type	T°^a^	Diameter (in)		
			.014	.016	.019X.025
Dentaurum					
Rematitan® Lite	NiTi classico		•	•	
Tensic®	NiTi termico	30	•	•	•
Forestadent					
Titanol®-Superelastic	NiTi classico		•	•	
Biostarter®	NiTi termico	37	•	•	•
Ormco					
Align SE®	NiTi classico		•	•	•
Copper® NiTi	NiTi termico	27	•	•	•
G&H					
Orthoforce G4®	NiTi classico		•	•	•
Orthoforce M5®	NiTi termico	37	•	•	•
American Orthodontics					
NiTi Memory Wire®	NiTi classico		•	•	•
Copper NiTi Wire Tanzo®	NiTi termico	27	•	•	•

^a^T° indicates transition temperature range (TTR)

### In vivo aging

In vivo group included 92 wires: 42 traditional and 50 heat-activated wires. The wires were retrieved during the regular treatment visits of patients selected with the following criteria: (1) both sexes in the age group 10–50; (2) no history of diabetes mellitus DM and/or infective chronic diseases; (3) no history of orthodontic treatments; (4) with mild crowding C1 or medium/severe crowding C2, according to Little’s index [8]; and (5) for who it was planned a non-self-ligating technique treatment with 0.022 Praxis SFP Lancer and 0.010 stainless steel ligature. In C1 patients were retrieved 0.016 and 0.019X0.025 cross-section wires, in C2 patients were also retrieved the 0.014 cross-section wires. Each wire was placed intraorally for a period of 1 month. One graduate student was instructed to monitor all NiTi archwires insertion and retrieval appointments by means of a retrieval protocol: (a) name of patient; (b) date of archwire placement; (c) archwire brand, cross section, and dental arch of insertion; and (d) date of archwire removal.

### In vitro aging

**Fig. 1:**
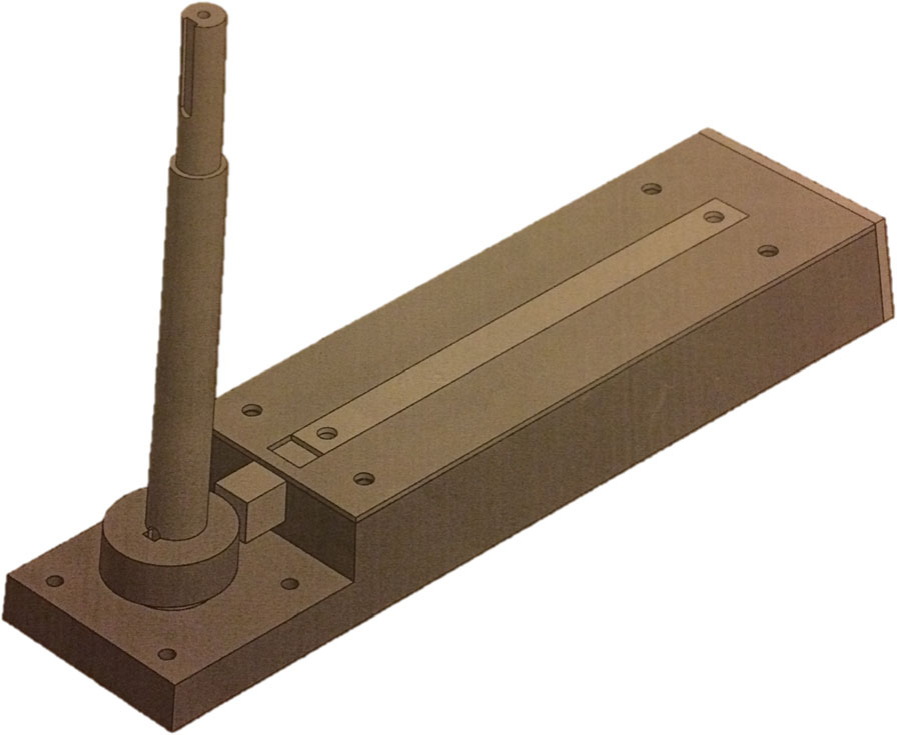
Stainless steel support

**Fig. 2:**
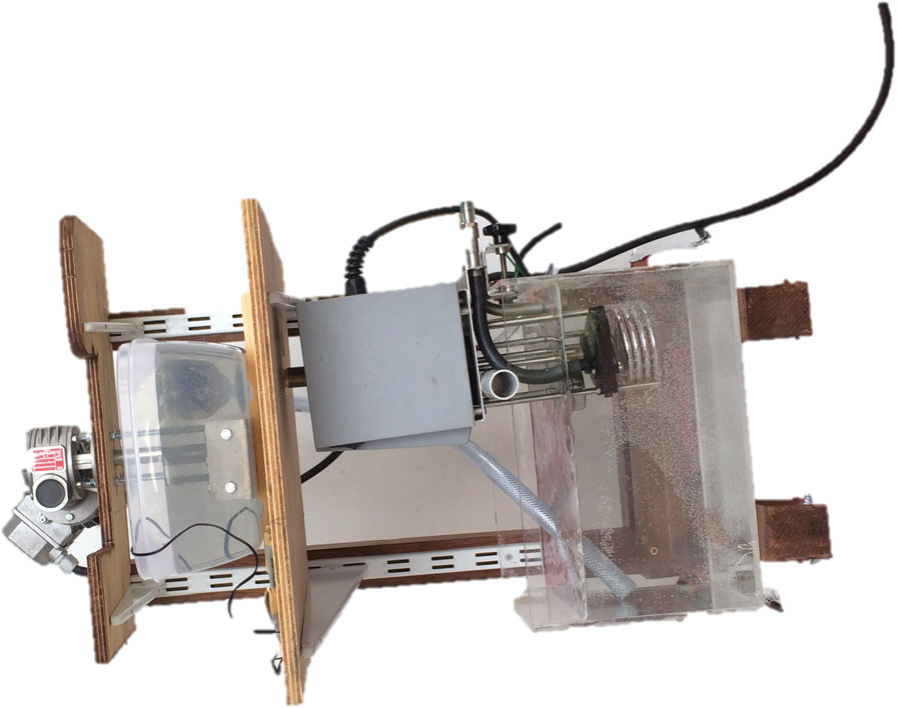
Aging machine composed by the gear motor, the stainless steel support, water and saliva baths, and the heating pump

In vitro group included 26 traditional and 30 heat-activated archwires. Three samples from each type of archwire of 5.5 cm lengths were obtained. For oral simulation, a stainless steel base (Fig. 1) with a mobile central part was connected to a gear motor (60 W power and 56 cycles per minute/1 Hz). On the base were bonded ten rows of brackets using metal glue. Each row contained five Edgewise Sinterline (Lancer, Italy) incisors, canines, and bicuspids brackets of 0.022-in slot size, which were positioned at the same line with their long axis parallel and the canine brackets glued on the mobile part of the base. The wires specimens were held on brackets by using 0.010 stainless steel ligatures. The base was placed in a plastic container filled with distilled water and artificial saliva (Biotene Oral Balance®) kept at a constant temperature of 37 °C [9–11] maintained with the aid of a heating pump (Julabo) placed in a separate water bath connected to the first by an hydraulic circuit (Fig. 2). Water temperature was controlled by means of a thermocouple (Tekkal T8303, Tekkal, Milan, Italy) submerged in the first bath and was monitored continuously by the same operator responsible for performing the aging test. Turning on the gear motor, the mobile part of the base moved deflecting each wires of 4 mm and then was returned to its horizontal starting point for a total of 100,000 cycles [12, 13].

### Three-point bending test

**Fig. 3:**
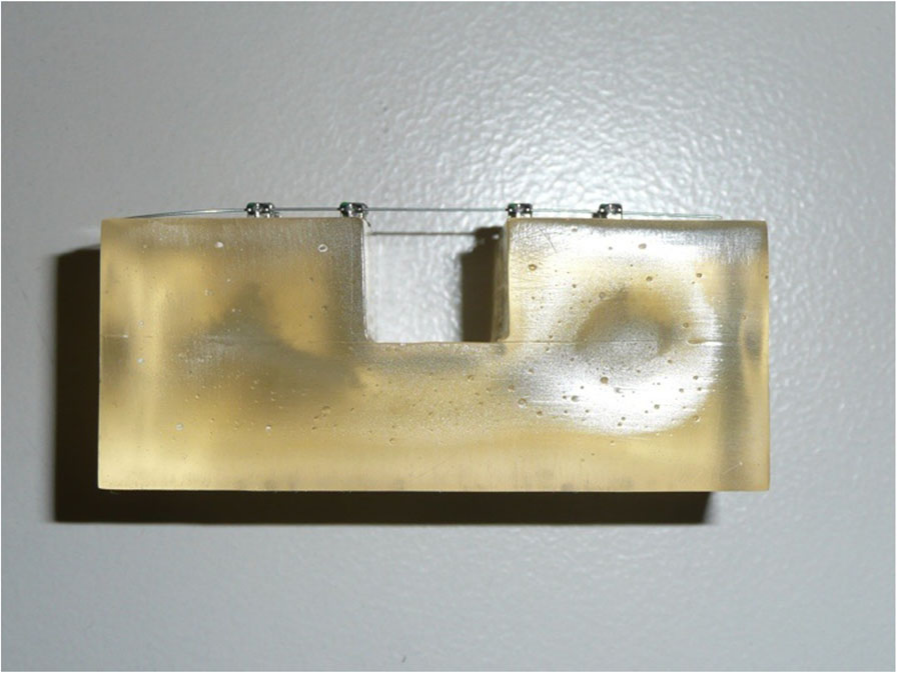
Mounted archwire on the acrylic resin base

Tests were performed on three samples of 5.5 cm lengths obtained by each archwire of the three conditions: retrieved in vivo, retrieved in vitro, and as received. They were tested in a three-point bending experiment [14–16]. To evaluate the samples under conditions similar to the final operating one, they were mounted in four non self-ligating brackets (0.022 Edgewise standard, Lancer) using 0.010 stainless steel ligatures to bend the wires to the brackets [9, 17]. The brackets were glues to an acrylic resin base [18] (Fig. 3) in such a way to create a 14-mm span between the internal sides of two adjacent brackets [19]. The resin base was, in turn, placed in a Plexiglas bath filled with distilled water and artificial saliva (Biotene Oral Balance®) kept at a constant temperature of 37 °C. The temperature of 37 °C was maintained with the aid of a heating pump (Julabo, Julabo Labortechnik Gmbh, Seelbach, Germany) placed in a separate bath connected to the first one by a hydraulic circuit. Temperature was controlled by means of a thermocouple (Tekkal T8303, Tekkal, Milan, Italy) submerged in the test bath and was monitored continuously by the same operator responsible for performing the mechanical tests. The force applied was regulated by means of an Instron 4467 dynamometer (Instron, Norwood, Mass) connected to a 100-N load cell. A metal blade, with a curvature range of 1 mm at its extremity, was fixed to the load cell to deflect the archwires. Each wire was deflected 4 mm [4, 9], at a deflection speed of 1 mm/min, and then was returned to its horizontal starting point at the same speed.

### Measurements

Data were gathered by means of a personal computer connected to the measuring device and were processed using Labview 8.5 (National Instruments Corporation, Austin, Texas). Data thereby collected were presented in spreadsheet form using Microsoft Excel (Microsoft Corporation, Redmond, Wash) and then used to plot a graph for each test, showing deflection of the test strip on the *x*-axis and the force exerted on the *y*-axis. Each curve thereby obtained represented the initial loading phase, of no particular clinical relevance, and the discharge phase, which indicates the entity of the force exerted on the teeth during orthodontic treatment. A sole operator subjectively identified and isolated on each graph the discharge plateau. We then characterized the behavior of the archwires in the plateau phase [4, 9] by measuring three parameters: average plateau force, plateau length, and plateau slope. The plateau length indicated the extension of the displacement range in which the force may be considered approximately constant. The average force was given by the arithmetic average of the force values pertaining to the discharge phase. The effective slope is a measure of the degree of plateau flatness; therefore, the closer was to zero, the more constant was the force [4, 9]. A load/deflection curve was obtained for each of the samples of each type of wire tested and the values yielded by the samples for each of the parameters considered were calculated [4, 9].

### Statistical analysis

Statistical analysis of the data was performed with the classification and regression trees (CART) using the implementation provided by the *party* R package. The CART is a statistical method belonging to the Data Mining family, frequently used when there are many variables to relate with respect to a depended one. It shows recursive splits that hierarchically explain the dependency relationship partitioning data into splits determined by the level of available predictors that recursively better explain the relationship between dependent and independent variables, which can be either numeric or categorical. It allows to easily uncover independent variables’ interaction as well as the hierarchy between variables. Its output, a set of if-then rules, can be also expressed graphically displaying a “tree” of variables’ dependency. Splits are determined using non-parametric statistical tests (“permutation tests” as better detailed in the cited material) for which *p* values are reported. The used technique does not rely on parametric assumptions (as the ANOVA’s normality one) and this is one of the reasons for which a retrospective sample size assessment is not available. The analysis related the variables plateau force, length, and slope with the factors type of wires (traditional and heat activated), size of the wires, brands, and conditions (wires retrieved in vivo, retrieved in vitro and as received). The CART, in the package party [20] of the statistical software R [21], was used to relate all the data bc of the great number of factors. Finally, inter-rater reliability has been assessed performing three replicates of the same measure and calculating the ICC statistic [22].

## Results

The analysis was focused on differences in the parameters examined between as received and retrieved NiTi archwires (Tables 2 and 3).

**Table 2:** The mean values yielded by as received and retrieved traditional orthodontic wires

Manufacturer		As received	SD	In vitro	SD	In vivo 1	SD	In vivo 2	SD
Dentaurum	Plateau force, g_f_	147.8	3.9	173.4	9.1			141.2	13.9
0.014	Plateau slope, g_f_/mm	− 2.8	1.7	4.2	2.3			3.4	2.3
	Plateau length, mm	1.7	0.4	1.4	0.3			1.3	0.2
Dentaurum	Plateau force, g_f_	222.9	11.9	204.0	63.6	225.1	1.5	184.3	62.9
0.016	Plateau slope, g_f_/mm	− 6.4	4.0	9.5	4.2	4.4	2.0	6.2	5.2
	Plateau length, mm	1.4	0.3	1.4	0.1	1.4	0.1	1.6	0.3
Forestadent	Plateau force, g_f_	124.3	16.3	123.0	11.2			131.4	24.9
0.014	Plateau slope, g_f_/mm	4.4	2.1	5.8	7.0			1.86	0.5
	Plateau length, mm	1.6	0.8	1.7	0.2			1.9	0.8
Forestadent	Plateau force, g_f_	211.2	5.3	198.9	15.5	239.3	15.7	260.0	4.8
0.016	Plateau slope, g_f_/mm	5.9	1.6	− 2.9	6.8	4.5	10.9	9.4	3.5
	Plateau length, mm	1.1	0.3	1.5	0.1	1.3	0.1	1.3	0.3
Ormco	Plateau force, g_f_	110.0	7.0	60.7	30.3			128.7	4.1
0.014	Plateau slope, g_f_/mm	− 3.4	1.9	0.5	3.7			1.7	3.2
	Plateau length, mm	1.7	0.4	1.7	0.6			1.4	0.2
Ormco	Plateau force, g_f_	159.7	9.0	131.7	46.1	173.0	21.7	131.3	44.7
0.016	Plateau slope, g_f_/mm	− 11.4	6.0	− 4.6	6.8	− 17.8	8.8	− 10.3	9.4
	Plateau length, mm	2.1	0.4	1.9	0.3	1.9	0.2	1.7	0.5
Ormco	Plateau force, g_f_	615.6	17.3	618.7	24.5	490.8	282.8	646.1	78.9
0.019X0.025	Plateau slope, g_f_/mm	20.3	32.9	28.8	22.3	14.0	26.7	49.2	45.9
	Plateau length, mm	1.6	0.2	1.5	0.2	2.3	0.2	2.0	0.2
G&H	Plateau force, g_f_	125.0	2.1	124.8	7.6			106.8	2.7
0.014	Plateau slope, g_f_/mm	2.7	1.0	3.3	5.1			6.4	6.0
	Plateau length, mm	0.9	0.5	1.7	1.0			1.3	0.4
G&H	Plateau force, g_f_	159.1	2.5	187.5	10.3	173.7	6.0	167.5	6.5
0.016	Plateau slope, g_f_/mm	− 5.9	3.8	8.8	8.3	5.7	6.6	10.2	5.6
	Plateau length, mm	1.8	0.2	1.3	0.4	1.4	0.5	1.7	0.4
G&H	Plateau force, g_f_	697.4	3.3	636.4	207.7	581.4	122.3	687.2	4.6
0.019X0.025	Plateau slope, g_f_/mm	28.2	6.9	51.3	23.6	− 9.1	61.6	41.2	24.5
	Plateau length, mm	2.2	0.1	2.3	0.3	1.9	0.2	1.9	0.3
American Orthodontics	Plateau force, g_f_	133.3	2.0	169.6	8.2			118.7	31.3
0.014	Plateau slope, g_f_/mm	− 1.8	2.3	5.8	2.5			− 2.8	1.8
	Plateau length, mm	0.9	0.2	1.4	0.4			1.1	0.5
American Orthodontics	Plateau force, g_f_	216.7	4.0	262.6	26.1	152.0	14.9	180.0	70.4
0.016	Plateau slope, g_f_/mm	− 2.5	5.7	16.2	6.7	− 25.7	15.9	0.1	10.6
	Plateau length, mm	1.7	0.4	1.3	0.2	0.8	0.1	1.4	0.3
American Orthodontics	Plateau force, g_f_	674.1	21.8	806.2	8.0	618.7	87.7	705.9	63.3
0.019X0.025	Plateau slope, g_f_/mm	34.7	17.7	61.0	20.7	− 11.9	47.7	51.1	25.5
	Plateau length, mm	2.2	0.1	2.0	0.2	1.8	0.3	1.6	0.2

**Table 3:** The mean values yielded by as received and retrieved heat-activated orthodontic wires

Manufacturer		As received	SD	In vitro	SD	In vivo 1	SD	In vivo 2	SD
Dentaurum	Plateau force, g_f_	101.1	1.4	123.4	6.5			116.5	19.9
0.014 T°	Plateau slope, g_f_/mm	4.0	1.3	6.2	1.2			6.1	7.8
	Plateau length, mm	1.4	0.3	1.3	0.2			1.4	0.3
Dentaurum	Plateau force, g_f_	93.8	2.9	118.4	7.7	82.3	16.3	103.8	7.4
0.016 T°	Plateau slope, g_f_/mm	8.4	0.7	8.9	1.7	−4.1	10.0	6.51	0.8
	Plateau length, mm	1.8	0.3	1.6	0.2	1.8	0.5	1.7	0.3
Dentaurum	Plateau force, g_f_	378.2	18.4	277.4	13.1	337.2	114.2	451.5	8.0
0.019X0.025 T°	Plateau slope, g_f_/mm	52.3	15.0	52.8	8.2	43.8	21.5	58.6	15.6
	Plateau length, mm	2.1	0.3	2.0	0.2	1.9	0.2	1.5	0.3
Forestadent	Plateau force, g_f_	95.2	0.5	90.3	2.5			116.5	4.7
0.014 T°	Plateau slope, g_f_/mm	11.7	3.6	6.3	2.4			3.13	1.9
	Plateau length, mm	1.2	0.3	1.9	0.2			2.1	0.7
Forestadent	Plateau force, g_f_	130.7	1.0	135.6	5.9	129.4	17.7	93.5	43.6
0.016 T°	Plateau slope, g_f_/mm	5.4	1.2	3.7	3.8	0.7	9.4	3.8	6.9
	Plateau length, mm	1.2	0.2	1.2	0.2	1.3	0.4	2.0	1.3
Forestadent	Plateau force, g_f_	392.4	37.3	365.6	9.0	384.3	55.8	349.3	41.3
0.019X0.025 T°	Plateau slope, g_f_/mm	65.7	4.0	79.6	10.1	54.3	50.9	46.5	13.9
	Plateau length, mm	1.8	0.1	1.9	0.2	2.1	0.3	1.8	0.3
Ormco	Plateau force, g_f_	126.0	5.5	112.6	36.2			145.8	8.4
0.014 T°	Plateau slope, g_f_/mm	− 3.7	3.4	3.8	14.6			− 2.3	3.6
	Plateau length, mm	0.9	0.1	1.4	0.1			1.6	0.4
Ormco	Plateau force, g_f_	163.8	10.5	144.9	31.9	163.2	22.2	160.3	14.4
0.016 T°	Plateau slope, g_f_/mm	− 6.9	2.5	13.9	1.1	− 11.1	13.7	− 8.9	6.0
	Plateau length, mm	1.6	0.5	0.9	0.2	1.9	0.2	1.2	0.4
Ormco	Plateau force, g_f_	480.7	41.2	559.9	24.1	467.9	101.2	466.1	41.6
0.019X0.025 T°	Plateau slope, g_f_/mm	12.6	37.0	58.6	43.7	− 36.2	75.1	− 17.6	8.8
	Plateau length, mm	1.6	0.2	1.9	0.4	2.4	0.4	2.7	0.2
G&H	Plateau force, g_f_	65.3	2.2	43.1	3.6			47.6	2.1
0.014 T°	Plateau slope, g_f_/mm	3.7	2.0	6.6	0.6			9.38	3.0
	Plateau length, mm	1.6	0.2	1.5	0.1			1.2	0.5
G&H	Plateau force, g_f_	87.0	1.7	58.5	8.8	85.7	11.6	71.1	16.4
0.016 T°	Plateau slope, g_f_/mm	9.1	2.1	8.5	5.3	11.0	2.1	17.7	4.3
	Plateau length, mm	1.9	0.3	1.7	0.4	2.1	0.1	1.4	0.3
G&H	Plateau force, g_f_	326.6	14.6	266.5	6.6	161.4	150.3	296.0	21.1
0.019X0.025 T°	Plateau slope, g_f_/mm	36.8	25.2	48.5	8.0	8.4	46.8	54.7	5.6
	Plateau length, mm	2.6	0.3	1.9	0.2	2.3	0.6	2.0	0.2
American Orthodontics	Plateau force, g_f_	107.3	22.5	156.7	2.4			152.3	64.2
0.014 T°	Plateau slope, g_f_/mm	− 0.3	2.2	5.5	3.6			5.4	6.4
	Plateau length, mm	2.2	0.4	1.3	0.1			1.2	0.2
American Orthodontics	Plateau force, g_f_	168.7	1.4	209.3	7.0	154.0	13.8	142.2	18.8
0.016 T°	Plateau slope, g_f_/mm	− 2.6	1.5	7.8	5.0	− 5.2	5.9	− 1.3	1.3
	Plateau length, mm	2.0	0.1	1.8	0.4	1.7	0.3	1.8	0.3
American Orthodontics	Plateau force, g_f_	490.6	9.5	533.9	10.4	316.3	28.8	175.6	45.6
0.019X0.025 T°	Plateau slope, g_f_/mm	22.3	0.8	75.6	18.3	− 30.4	7.8	− 20.4	32.9
	Plateau length, mm	2.6	0.1	3.0	1.8	1.5	0.7	1.4	0.6

### Plateau force

For all the brands, with some exceptions, the force becomes greater with the increase of the wire size. A diminish of force was registered in the heat-activated wires when compared to the traditional ones, also with some exceptions. This trend is followed by as received, retrieved in vitro, and in vivo wires. These differences in size and type of wire, though, were not statistically significant. The ICC statistic for intra-rater reliability is 0.98.

### Plateau length

The plateau length increases with the increase of the wire size: the shortest plateaus were represented by 0.014 wires, where the longest plateaus were represented by 0.019X0.025 wires, with few exceptions. This trend is shared by all the three different conditions: as received, retrieved in vitro, and in vivo wires. These differences, though, were not statistically significant. The ICC statistic for intra-rater reliability is 0.65.

### Plateau slope

The plateau slope is higher in 0.019X0.025 wires compared to the other two wire sizes tested, with some exceptions. This trend is followed by all the wires, even if it was registered a higher variability of the data in the in vitro retrieved wires and in vivo retrieved moderate/severe group (C2) wires. The differences registered in slope were statistically significant. The ICC statistic for intra-rater reliability is 0.81.

## Discussion

Orthodontic materials in the oral cavity might not perform identically to their as received or in vitro aged counterparts, and their properties might deviate from those specified by the manufacturer [5, 6]. Nakano et al. [19] showed that different brands of NiTi alloy wires of the same size varied widely in the force levels they exerted. During orthodontic treatment, occlusal contact between the dental arches, swallowing, and chewing result in forces that are transferred to the archwires. Repeated occlusal contact combined with tooth mobility, which occurs during alignment, might compromised the performance and the efficiency of the deflected archwire [13]. There is a scarcity of published researches on in vivo aged orthodontic wires mechanical properties [5, 6]. In vitro-retrieved protocol can be static or dynamic [13]. In this study, we built an in vitro-retrieved dynamic protocol in order to recreate a situation the closest possible to clinical conditions. The dynamic protocol was performed by an aging machine completely designed by the Engineering Department and the Orthodontic Department of the University of Ferrara. The aging machine simulates a patient’s archwire under deflection and subjected to intermittent occlusal contact. It represents 7 weeks (100,000 cycles) [13] of in vivo behavior, approximately the recall time to check the progress of tooth alignment. The wires were retrieved in vitro at 37 °C, which represents the intraoral temperature, in artificial saliva [19, 23, 24]. The same condition was maintained for the three-point bending test. It was chosen to deflect the wires 4 mm because it was found that with a small deflection (0.5 mm), there was no SIM (martensitic transformation induced by stress), instead at greater deformations a superelastic behavior was exhibited by the wires. The three-point bending test was used to test the mechanical behavior of the wires as received, retrieved in vitro, and retrieved in vivo. Only data from the unloading portion of the load-deflection curve were reported because these are the forces actually distributed to the teeth by orthodontic wires during treatment (working forces).

### NiTi wires mechanical behavior

The force plateau is influenced by all the factors analyzed in this study except for the condition. The force expressed depends on the type of wire: heat-activated wires tend to impart less force than traditional NiTi archwires. In addition, greater force is shown by increasing the size of wires, as previously demonstrated by Lombardo et al. [9]. Our study revealed that the force depends even on wires brands. It was registered a reduce force for 0.014 and 0.016 heat-activated wires of the brands Dentaurum, Forestadent, and G&H. Same result was reported in Lombardo et al.’s [9] study. The plateau length is influenced only by the size wire factor; the condition does not influence the plateau length. The 0.019X0.025 wires showed the highest plateau length when compared to the sizes 0.014 and 0.016. Lombardo et al. [9] analyzed only round as received archwires so it was not possible to compare this data. The force and length plateau parameters could be compared to Lombardo et al.’s [9] mechanical behavior wires results because it is the only study in literature to analyze these parameters and the force and length were shown, by this study, to not being influenced by the condition. The plateau slope is the only parameter to be influenced by the condition. The plateau slope expresses the capacity of an archwire to exert more constant forces with increasing displacement [4, 9]. This means that the condition can modify the performance of the wire in term of dental movement. The 0.019X0.025 wire retrieved in vivo C1 and as received showed a lower slope when compared to the other conditions. Same behavior was registered for the traditional 0.014 and 0.016 wires of three brands and both traditional and heat-activated 0.014 and 0.016 wires of two brands. Our study is in accordance with Zinelis et al. [25] that demonstrated no increase in the hardness of the intraorally exposed specimens when compared to the as received. Moreover, it shows that wires do not improve their performance with the increase of the deflection in fact retrieved in vivo C2 wires showed a worse performance in term of mechanical behavior. The clinician will then modify on the everyday practice the patient recall during the orthodontic treatment depending on the crowding, brand, and size of the wire chosen.

### Study limitations

The in vitro retrieval protocol was designed in order to retrieve archwires by cycles of deflections at a constant temperature. Further researches should outline a protocol that will retrieve wires through a deflecting and thermocycling regimen. It was demonstrated that thermocycling regimen comprising a minimum of 500 cycles in water between 5° and 55 °C is an appropriate artificial aging test [26]. Although the deflection chosen, 4 mm, represents partially the great variability of in vivo dental crowding. Four millimeter is considered a norm deflection in the oral environment [13]. In further studies, it could be interesting to design an aging machine for each in vivo crowding groups (C1 and C2), changing the deflection.

## Conclusions

All NiTi wires tested, as received and retrieved, showed a mechanic behavioral change only for the parameter slope (force constancy). The condition affects only the force constancy but does not affect the force expressed by the NiTi wires and the displacement range in which the force is constant.

The wires 0.019X0.025 as received and in vivo retrieved light crowding group (C1) expressed a more constant force.

The wires 0.014 and 0.016 traditional as received and in vivo retrieved C1 of three brands registered a higher constant force.

The wires 0.014 and 0.016, traditional and heat-activated, as received, and in vivo retrieved C1 of two brands showed a greater force constancy.
